# Patterns of Childhood Cancer Mortality in Hungary Since the Turn of the Millennium, Including the Two Years of the COVID-19 Pandemic

**DOI:** 10.3390/cancers16233961

**Published:** 2024-11-26

**Authors:** Kristóf Németh, Tibor András Nyári, Tamás Lantos

**Affiliations:** 1 Biological Institute, University of Szeged, Közép fasor 52, H-6720 Szeged, Hungary; 2 Department of Medical Physics and Informatics, University of Szeged, Korányi fasor 9, H-6720 Szeged, Hungary; lantos.tamas@med.u-szeged.hu

**Keywords:** childhood cancer mortality, regional differences, COVID-19 pandemic, double-peak seasonality, geometric models, trends, Hungary

## Abstract

**Objectives**: We aimed to analyse the pattern of childhood cancer mortality among children under 15 years in Hungary between 2001 and 2021. In addition, annual and cyclical trends were examined. **Methods**: The number of deaths was obtained from the nationwide population register over the study period by gender, age group, and region for each year. Data were analysed using the Poisson regression method and cyclic trends were investigated using the Walter–Elwood method. **Results**: Overall, 14,931 childhood deaths (1092 from cancers) were registered between 2001 and 2021. The cancer mortality risk was significantly higher among boys than girls. A significantly decreasing trend was detected for yearly childhood cancer mortality rates, with an annual IRR of 0.976 (95% CI: 0.966–0.986; *p* < 0.001). This tendency was not influenced by the pandemic. However, different patterns of seasonal variation were revealed in childhood cancer mortality rates during 2020–2021 and 2001–2019. **Conclusions**: The annual trend in childhood cancer mortality was not affected by the coronavirus pandemic. However, there was a different pattern of childhood cancer mortality during the pandemic and non-pandemic period in Hungary. Considering the seasonal variation in monthly childhood cancer mortality rates, we hypothesized that environmental factors might play an important role in the aetiology of childhood cancer deaths.

## Simple Summary

During the COVID-19 period, health care had to be cut back, which may also have affected the numbers and patterns of childhood cancer deaths in Hungary. We aimed to analyse the pattern of childhood cancer mortality among children under 15 years. The decreasing annual trend in childhood cancer mortality was not affected by the coronavirus pandemic. However, there was a different pattern of childhood cancer mortality during the pandemic and non-pandemic period in Hungary.

## 1. Introduction

Some studies have reported that the time trend in survival after childhood cancer in Europe was highest in the north and the west and lowest in the east, and that this ranking among the European regions has not changed in recent decades [1,2,3].

Despite the outcome of childhood cancers showing an improvement and the average survival time of patients increasing, the Hungarian mortality rate of all neoplasms and leukaemia is still high in Europe [4].

In addition, a highly elevated risk of death in the second 5 years of follow-up was reported for late mortality in survivors of childhood cancer in Hungary [5].

Centralised treatment protocols and national registration for children with leukaemia and solid tumours were introduced by centres of the Hungarian Paediatric Oncology Network (HPON) in 1971 [5]. Patients are treated in paediatric oncology centres according to uniform guidelines. The individual centres are in Budapest and five other regional centres of the country. However, no studies have been carried out to investigate regional differences.

The seasonality of disease suggests some effect of environmental factors in aetiology (e.g., infections). Environmental effects were reported in the aetiology of childhood acute leukaemia [6,7]. However, the cyclical trend in cancer mortality is rarely investigated.

In the years of the COVID-19 pandemic, a reduction in the number of newly diagnosed paediatric tumours, probably reflecting the delayed access to healthcare services for patients with symptoms suspected to be cancer-related, was observed [8].

The present ęcological study was performed to determine regional differences and both the annual and cyclic trends in the mortality rate of cancer death among children under the age of 15 years in Hungary during the 21-year interval between 1 January 2001 and 31 December 2021, which includes the two years of the COVID-19 pandemic period.

## 2. Materials and Methods

### 2.1. Study Population

The analysis considered a 21-year period from 2001 to 2021. Data on the cause of death were classified according to the 10th revision of the International Classification of Diseases (ICD-10) codes. Annual data on the number of deaths at age 0–14 years due to all neoplasms (ICD-10: C00–D48) were extracted from the nationwide population register maintained by the Hungarian Central Statistical Office (HCSO) [9]. The HCSO provides data on the number of deaths over the study period by gender, age group, and region for each year.

Male-to-female ratios were calculated as the ratio of the incidence rate for males relative to that for females. Both the number of deaths from cancers and the population data were available broken down by the following age groups: 0–4 years, 5–9 years, and 10–14 years.

Similarly, data on the monthly numbers of deaths from childhood cancers were also obtained from the HCSO. However, no monthly data on the childhood population aged 0–14 years were available. Therefore, we have used annual population data and the monthly birth and death data to estimate the number of people in this age group by month.

Monthly mortality rates for childhood cancer were calculated as the number of deaths divided by the population in the same month of the year. The proportionate childhood cancer mortality rate was calculated as the fraction of all deaths under 15 years due to a specific cause.

**Figure 1:**
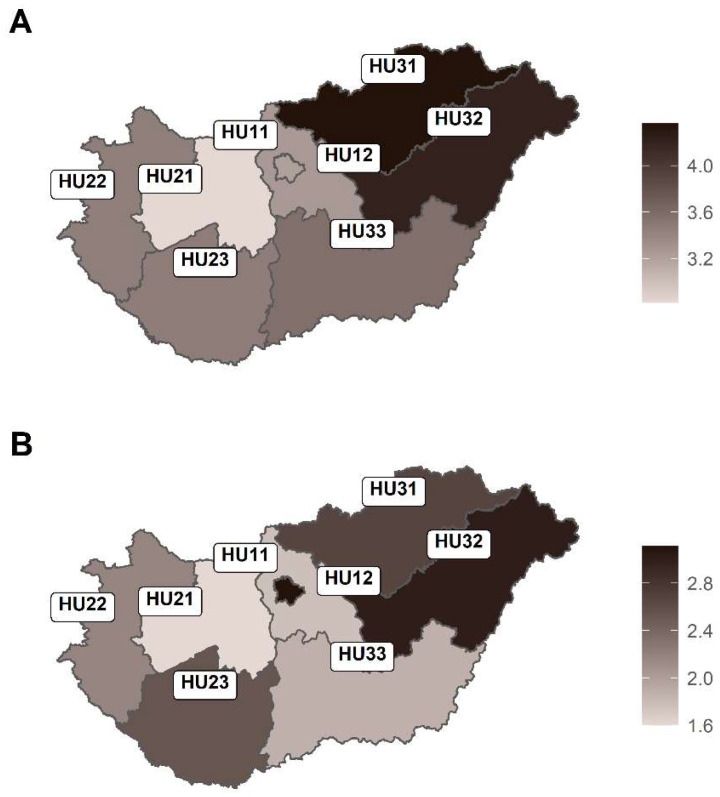
NUTS2 regions of Hungary coloured by age-standardised childhood cancer mortality rates per 100,000 children in the periods (**A**) 2001–2019 and (**B**) 2020–2021. Codes: *HU11*—Budapest; *HU12*—Pest County, Central Hungary; *HU21*—Central Transdanubia; *HU22*—Western Transdanubia; *HU23*—Southern Transdanubia; *HU31*—Northern Hungary; *HU32*—Northern Great Plain; *HU33*—Southern Great Plain. The maps depicted in the figure are the authors’ own work and were created using R, packages *sf* (version 1.0-12; https://cran.r-project.org/web/packages/sf/index.html, accessed on 25 September 2024 ) and *ggplot2* (version 3.3.5; https://cran.r-project.org/web/packages/ggplot2/index.html, accessed on 25 September 2024).

Territorial units were based on the second level of the NUTS 2016 (*Nomenclature des unités territoriales statistiques*—Nomenclature of Territorial Units for Statistics, 2016 revision) classification [10]. These statistical regions (second level of the classification; NUTS2) were as follows (Figure 1): Budapest (the capital region—formerly part of Central Hungary; HU11), Pest County (the surrounding region—formerly part of Central Hungary; HU12), Central Transdanubia (HU21), Western Transdanubia (HU22), Southern Transdanubia (HU23), Northern Hungary (HU31), Northern Great Plain (HU32), and Southern Great Plain (HU33).

### 2.2. Statistical Methods

Mortality rates were expressed per 100,000 population per year using the annual mid-year population estimates for the relevant year. Age-standardised mortality rates (ASMRs) in Hungary during the study period were calculated according to the direct method [11] using the Revised European Standard Population (RESP) [12] for children aged under 15 years (in three age groups: 0–4, 5–9, and 10–14 years) to make regional mortality rates comparable over time.

Data on the month of death were aggregated over the study period and cyclic trends were analysed using the Walter–Elwood and the Poisson regression methods [13,14]. Both methods adjust for the population at risk by grouping the data into months and were used to investigate single or double peaks of seasonality. In contrast, the geometrical model by Edwards [15], which does not take the population into account, was used as a sensitivity analysis to control for population effects on the cyclical trend.

Trends in the annual numbers of deaths were also investigated using Poisson regression. Incidence rate ratios (IRRs) were calculated together with their corresponding 95% confidence intervals (95% CIs). The main requirement of this model is that the variance of the dependent variable is equal to its mean (“equidispersion”). Since the likelihood ratio test for equidispersion was non-significant, the model assumptions were fulfilled. In a sensitivity analysis, joinpoint regression was also applied to describe the tendency in annual childhood cancer mortality rates.

The death rate from childhood cancers during the two years of the COVID-19 pandemic (2020–2021) was compared using Poisson regression with the death rate from childhood cancers during the previous nearly two decades of the non-pandemic period between 2001 and 2019. Furthermore, to investigate cyclic trends, the non-pandemic study period between 2001 and 2019 was divided into two sub-periods: 2001–2010 and 2011–2019.

A *p*-value of less than 0.05 was taken to indicate a significant effect. All analyses were performed with the STATA software, version 17.0 (Stata Corp LP, College Station, TX, USA).

## 3. Results

Overall, 14,931 deaths were registered during the study period among children aged 0–14 years, of which 1092 (7.3%) were deaths from childhood cancer. The most frequent causes of death from childhood cancers were the following: 303 (27.7%) malignant tumours of the central nervous system (C71), 180 (16.5%) lymphoid leukaemia (C91), 92 (8.4%) myeloid leukaemia (C92) and 53 (4.9%) non-Hodgkin lymphoma (C82–C85, C88, C96). In addition, 66 (6%) deaths from childhood cancer occurred during the years of the coronavirus pandemic.

The childhood cancer death rate was significantly higher for boys, as there were 610 (56%) and 482 (44%) deaths in the groups of boys and girls, respectively (Table 1). The ratio of men to women was 1.27 (95% CI: 1.13–1.143), which slightly decreased to 1.19 in the COVID-19 pandemic period.

During the study period, the age-standardised childhood cancer mortality rate (ASMR) was 3.49 (SE = 0.18) deaths per 100,000 children aged 0–14 years in Hungary; it was significantly (*p* = 0.02) higher in the non-pandemic period (3.57 per 100,000 children; SE = 0.19) than in the COVID-19 period (2.38 per 100,000 children; SE = 0.5). Similarly, ASMR was significantly (*p* = 0.02) higher in boys (3.80 deaths per 100,000 children and SE = 0.27) than in girls (3.16 deaths per 100,000 children and SE = 0.25). The risk of childhood cancer mortality was significantly lower in older age groups (5–9 years and 10–14 years) than in children aged under five years (IRR = 0.816; 95% CI: 0.756–0.879; *p* < 0.001). However, during the COVID-19 pandemic period, the risk of childhood cancer mortality was not significantly different in the age groups (IRR = 0.917; 95% CI: 0.685–1.227; *p* = 0.56).

Table 1 summarises the main characteristics of childhood mortality for those aged 0–14 years.

The territorial proportions of age-standardised deaths from childhood cancer varied significantly (*p* = 0.023), between 2.82 and 4.36 deaths per 100,000 children in the non-pandemic study period. The lowest and highest mortality rates were detected in the regions of Budapest (capital) and Northern Hungary/Northern Great Plain, respectively. The mortality rates remained high in the regions of Northern Hungary and Northern Great Plain during the coronavirus pandemic period in Hungary. However, during the COVID-19 pandemic period, the highest territorial mortality rate of 3.11 deaths per 100,000 children was observed in the Capital region in Hungary (Figure 1A,B). There were 82 (27%) of 303 deaths from CNS tumours in the regions of Northern Hungary and Northern Great Plain during the study period.

### 3.1. Annual Trends in Childhood Cancer Mortality Rates

**Figure 2:**
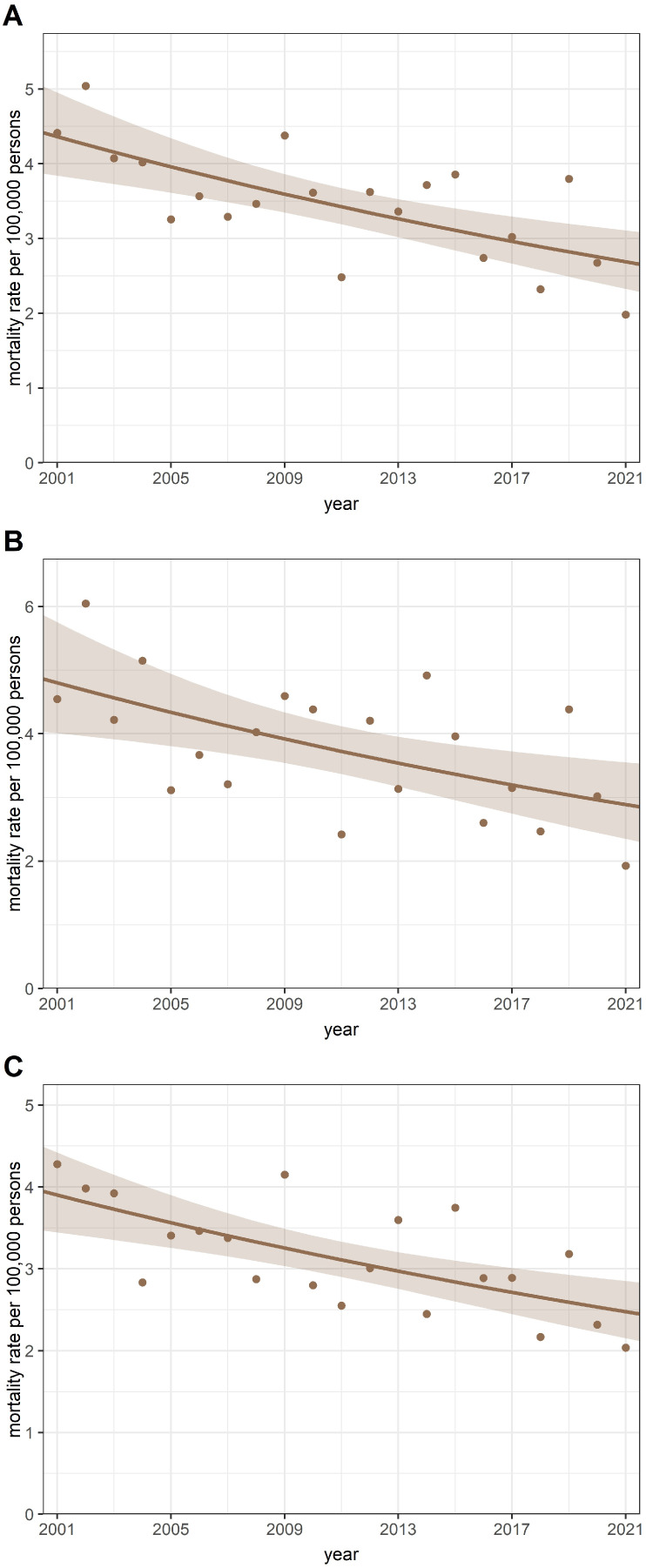
Annual trends in childhood cancer mortality rates. (**A**) All deaths from childhood cancer. (**B**) Male deaths from childhood cancer. (**C**) Female deaths from childhood cancer.

The crude annual mortality rate in children aged 0–14 years declined from a maximum of 66.82 per 100,000 children in 2001 to a minimum of 31.33 per 100,000 children in 2021, and there was a significant IRR trend per annum of 0.963 (95% CI: 0.961–0.966; *p* < 0.001). Furthermore, a significantly decreasing trend from a maximum of 5.04 per 100,000 children in 2002 to a minimum of 1.98 per 100,000 children in 2021 was detected for the annual rate of childhood cancer mortality, with an annual IRR of 0.976 (95% CI: 0.966–0.986; *p* < 0.001) (Figure 2A). A similar decreasing annual trend was observed in boys and girls during the study period in Hungary (Figure 2B,C). The findings of the joinpoint regression analysis confirmed that the coronavirus pandemic did not change the downward trend in childhood cancer mortality in Hungary.

### 3.2. Cyclic Trends in Childhood Cancer Mortality Rates

**Figure 3:**
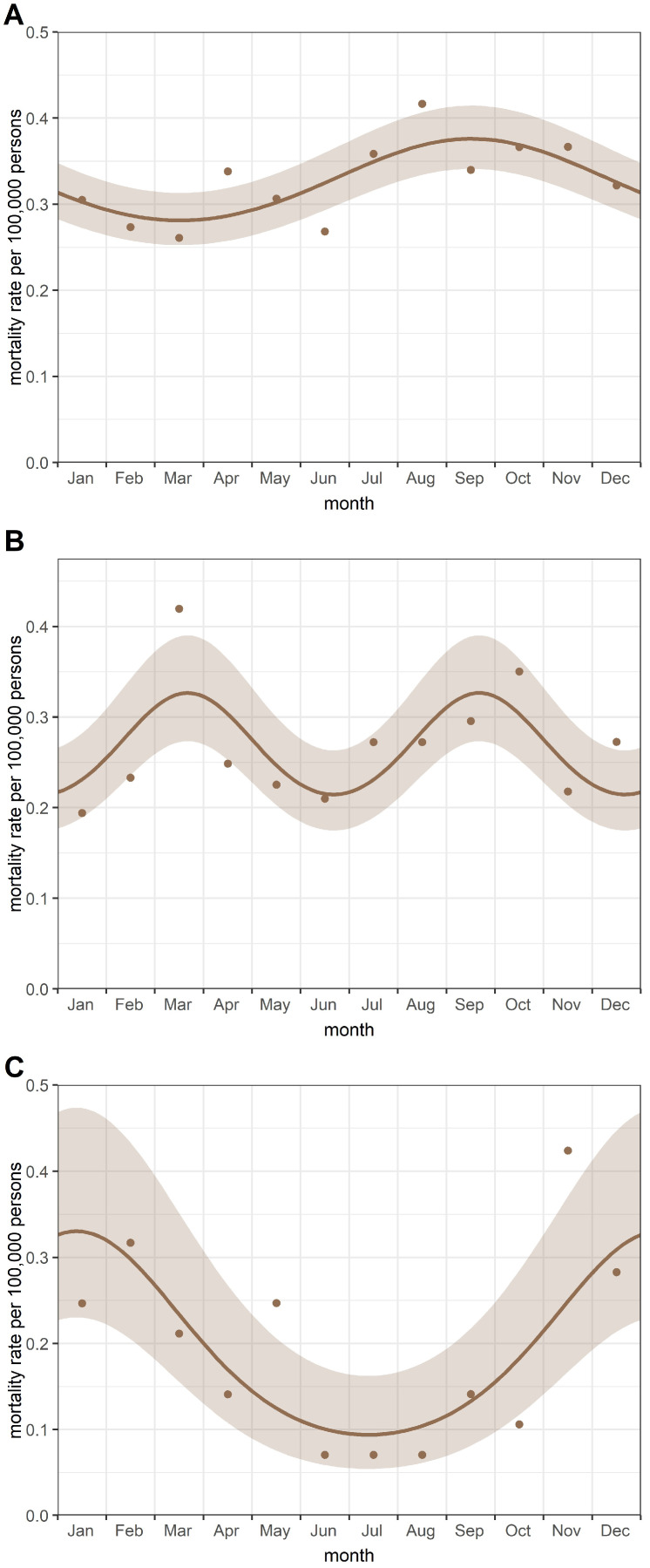
Cyclic trends in childhood cancer mortality rates in the periods (**A**) 2001–2010; (**B**) 2011–2019; and (**C**) 2020–2021.

No cyclic pattern was observed for the entire study period (i.e., between 2001 and 2021). A significant (*p* = 0.039) single peak in September was found in childhood cancer mortality rates in the period 2001–2010 (Figure 3A). In contrast, a double-peak model was fitted to characterise the seasonal variation in childhood cancer mortality between 2011 and 2019. The peaks were observed in March and September (*p* = 0.014) (Figure 3B). In addition, a significant (*p* < 0.001) single peak in January was observed in childhood cancer mortality rates during the coronavirus pandemic period (Figure 3C). All models had a reasonable goodness-of-fit statistic.

In the sensitivity analyses, the effect of the population on the seasonal variation was investigated. We applied the Edwards test without a population at risk and the Poisson regression using the proportional rate of childhood cancer mortality. The same cyclic trends were revealed using the Edwards test. Similar seasonal variations were also found in the proportionate childhood cancer mortality rates; however, the seasonal effect was not significant in the period between 2001 and 2010. The monthly death rate of children aged 0–14 did not show a cyclical trend during the study period. All models had a reasonable goodness of fit. The aggregate monthly numbers of deaths from childhood cancers between 2001 and 2021 in Hungary are summarised in Table 2.

## 4. Discussion

### 4.1. Main Findings

The ASMR for childhood cancer was 3.49 deaths per 100,000 children aged 0–14 years in Hungary; it was significantly higher in the non-pandemic period (3.57 per 100,000 children) than in the COVID-19 period (2.38 per 100,000 children). The risk of childhood cancer mortality was significantly higher among boys than among girls. Additionally, the risk of childhood cancer mortality was significantly lower in older age groups (5–9 years and 10–14 years) than in children aged under five years.

Changes in the spatial distribution of childhood cancer mortality were also observed in the years before and during the coronavirus pandemic. Although the mortality rate of childhood cancer remained high in the regions of Northern Hungary and Northern Great Plain, during the coronavirus pandemic, the capital (Budapest) had the highest regional mortality in Hungary.

A significant decrease in annual mortality rates for childhood cancers in those under 15 years was found in Hungary during 2001–2021. This trend was not influenced by the coronavirus pandemic.

However, different patterns of seasonal variation were detected in the mortality rates of childhood cancers during the two years of the COVID-19 pandemic and the preceding two-decade non-pandemic period. Significant seasonality was observed in monthly childhood cancer mortality rates, with peaks in September for the 2001–2010 period, March, and September for the 2011–2019 period, and January for the 2020–2021 period.

### 4.2. Comparison with Other Studies

The ASMR for childhood cancer was 3.49 deaths per 100,000 children aged 0–14 years in Hungary, which was similar to the ASMR of 3.56 per 100,000 children (aged 0–14) reported by Bertuccio et al. [4]. Similarly, our study also demonstrated that the mortality rates from all neoplasms were significantly higher in males than in females [16].

Although the mortality rate for childhood cancer remained high in the regions of Northern Hungary and Northern Great Plain, the capital had the highest regional mortality in Hungary during the coronavirus pandemic. The standard of living in Budapest is much higher than in the eastern regions of Hungary. The regions of Northern Hungary and Northern Great Plain are predominantly agricultural areas. Furthermore, in North-Eastern Hungary, Jakab et al. reported a higher incidence of central nervous system tumours in children [17]. The EUROCARE-5 study reported that childhood cancer survival time in Hungary was below the European average for both leukaemias and CNS tumours during the period 1999–2007 [18]. In a recent study, Jakab et al. [5] reported a similarly lower survival time than that reported in a French study [19]. Additionally, this study reported regional differences in cancer mortality among children under 15 years old [19]. Although we did not have morbidity data, our results showed that tumours of the central nervous system were the most common cause of childhood cancer deaths during the study period.

Childhood cancer death rates have declined in many developed countries [18]. Despite the downward trend, the Hungarian ASMR is still higher than in other EU countries [4].

Seasonal variation in cancer mortality is rarely investigated. In a previous study, Nyári and McNally also revealed no seasonal trend [20] in mortality rates of childhood cancers. Similarly, we have not found seasonality for the entire study period (2001–2021). However, we have revealed significant changes in the cyclical pattern of childhood cancer deaths in the sub-periods. A significant single peak in childhood cancer mortality rates was found in the periods from 2001 to 2010 and between 2020 and 2021 in Hungary, and double-peak seasonality was observed from 2011 to 2019. A significant peak in September was detected during the nearly two decades of the non-pandemic period between 2001 and 2019. We hypothesise that the effect of the “early autumn depression” might affect the increased accumulation of deaths from childhood cancers. According to several studies, seasonal affective disorder (SAD) can occur not only in winter but also in summer [21,22]. Therefore, the life expectancy of patients may deteriorate by the end of summer. In addition, Virág and Nyári described a significant cyclical variation in proportionate mortality for all cancer sites examined, with a peak in August or September in adults [23]. This suggests that the cancer death rate was higher at the end of August or at the beginning of September. In addition, higher average temperature changes were also observed in these months in Hungary [24].

Furthermore, the role of the respiratory syncytial virus (RSV) can also be assumed in the cyclical trend [25]. In a study, double peaks of RSV infection were reported in spring and autumn in temperate regions [25] and Bloom-Feshbach et al. found the peak of the flu epidemic and the RSV epidemic in the continental climate in the northern hemisphere was in February [26]. Studies from France, the United Kingdom, and Hungary described the role of infections around the time of birth in the aetiology of some childhood cancers [27,28,29,30]. However, the role of environmental effects in the aetiology of mortality from childhood cancers has not yet been investigated in detail.

During the years of the pandemic, the change in the cyclic trend can be clearly explained by active protection. Travel restrictions also reduced the number of infectious diseases brought home. In addition, patients were treated to a high standard and according to treatment protocols.

### 4.3. Strengths and Limitations of Our Study

Mortality data were obtained from the vital register of Hungary, which has one of the best statistics performance indices in the world [31]. The Hungarian Central Statistical Office applies the rules adopted by the World Health Assembly (WHA) regarding the selection of a single cause or condition, from death certificates, for the routine tabulation of mortality statistics to standardise the production of mortality data. Thus, there were no public data available on secondary cancers as a cause of death. Although trends in cancer mortality may be a combined result of incidence and survival, morbidity data were not available for all neoplasms in children aged 0–14 years.

Our study demonstrated significant regional and seasonal differences in the pattern of childhood all-cancer mortality in Hungary. However, there were no analyses carried out to differentiate mortality by cancer type. In this ęcological study, we have investigated the annual and seasonal pattern of mortality from childhood cancers in children aged 0–14 years. Although the number of events (deaths) was somewhat small in the ‘pandemic period’ (covering two years), the Walter–Elwood test is a powerful method in cases of extreme values.

Significant single-peak and significant double-peak models were observed to characterise the seasonal variation in mortality rates for childhood cancers. Despite the lack of direct evidence for respiratory syncytial virus infections in the analyses, we can speculate that these infections may play a role in the aetiology of mortality from childhood cancers [23]. Furthermore, the reliability of the fitted models was high. In addition, the investigation of seasonality is an important component in understanding the aetiological description of certain diseases.

Although seasonal patterns in all-cancer mortality have already been examined by other investigators, to the best of our knowledge, this is the first epidemiological study to report territorial differences and the effect of seasonality in mortality for childhood cancers in those aged 0–14 years in Hungary.

## 5. Conclusions

We provided a detailed description of seasonal effects related to mortality rates for childhood cancer deaths among children under 15 years. Although the annual trend in childhood cancer mortality was not affected by the coronavirus pandemic, there was a significant change in the mortality pattern of childhood cancer in Hungary during the two years of the COVID-19 pandemic and the preceding two-decade non-pandemic period. During the years of the pandemic, the cyclic trend in mortality from childhood cancer has also changed. Differences in the regional distribution of childhood cancer mortality were also observed by comparing periods before and during the pandemic.

Since cyclic trends in the aetiology of mortality may show some effect of environmental factors, we speculate that mortality from childhood cancer might have been (at least partly) related to respiratory infections. These findings could prove useful in preventive strategies, but further cohort studies should be carried out to investigate this hypothesis using detailed individual data.

## Figures and Tables

**Table 1:** Numbers of deaths (crude mortality rates/100,000 children) during 2001–2021 in Hungary by subgroup.

Subgroup	Childhood Cancer Deaths			All Childhood Deaths 2001–2021
	2001–2019	2020–2021	2001–2021	
GENDER				
Male	574 (3.93)	36 (2.47)	610 (3.80)	8418 (52.42)
Female	452 (3.26)	30 (2.18)	482 (3.16)	6513 (42.72)
AGE GROUP				
0–4 years	397 (4.42)	20 (2.13)	417 (4.20)	12,077 (121.71)
5–9 years	318 (3.39)	26 (2.83)	344 (3.34)	1228 (11.91)
10–14 years	311 (3.08)	20 (2.05)	331 (2.99)	1626 (14.69)
NUTS2 REGION				
Budapest (capital region)	138 (3.21)	14 (3.06)	152 (3.19)	1862 (39.65)
Pest (surrounding region)	126 (3.27)	8 (1.80)	134 (3.12)	1630 (37.97)
Central Transdanubia	87 (2.82)	5 (1.64)	92 (2.71)	1450 (42.75)
Western Transdanubia	92 (3.43)	6 (2.15)	98 (3.31)	1278 (43.14)
Southern Transdanubia	91 (3.50)	6 (2.52)	97 (3.41)	1395 (49.10)
Northern Hungary	159 (4.34)	9 (2.67)	168 (4.20)	2542 (63.48)
Northern Great Plain	198 (4.19)	13 (2.97)	211 (4.09)	2629 (50.92)
Southern Great Plain	128 (3.54)	6 (1.79)	134 (3.39)	1952 (49.38)

**Table 2:** Monthly numbers of deaths between 2001 and 2021 in Hungary.

Month	Childhood Cancer Deaths				All Childhood Deaths 2001–2021
	2001–2010	2011–2019	2020–2021	2001–2021	
Jan	48	25	7	80	1256
Feb	43	30	9	82	1178
Mar	40	54	7	101	1282
Apr	53	31	4	88	1265
May	48	30	7	85	1235
Jun	42	28	3	73	1216
Jul	56	36	3	95	1323
Aug	65	34	1	100	1210
Sep	54	39	2	95	1142
Oct	57	45	3	105	1370
Nov	57	28	12	97	1227
Dec	50	33	8	91	1227
*p*-value	0.039	0.014	<0.001	0.18	0.95
Peak(s)	Sep	Mar, Sep	Jan	NS *	NS *

* NS: Not significant.

## Data Availability

The data presented in this study are available in this article.
